# Utilizing Targeted Mass Spectrometry to Demonstrate Asf1-Dependent Increases in Residue Specificity for Rtt109-Vps75 Mediated Histone Acetylation

**DOI:** 10.1371/journal.pone.0118516

**Published:** 2015-03-17

**Authors:** Yin-Ming Kuo, Ryan A. Henry, Liangqun Huang, Xu Chen, Laurie A. Stargell, Andrew J. Andrews

**Affiliations:** 1 Department of Cancer Biology, Fox Chase Cancer Center, Philadelphia, Pennsylvania, United States of America; 2 Department of Biochemistry and Molecular Biology, Colorado State University, Fort Collins, Colorado, United States of America; Ludwig-Maximilians-Universität München, GERMANY

## Abstract

In *Saccharomyces cerevisiae*, Rtt109, a lysine acetyltransferase (KAT), associates with a histone chaperone, either Vps75 or Asf1. It has been proposed that these chaperones alter the selectivity of Rtt109 or which residues it preferentially acetylates. In the present study, we utilized a label-free quantitative mass spectrometry-based method to determine the steady-state kinetic parameters of acetylation catalyzed by Rtt109-Vps75 on H3 monomer, H3/H4 tetramer, and H3/H4-Asf1 complex. These results show that among these histone conformations, only H3K9 and H3K23 are significantly acetylated under steady-state conditions and that Asf1 promotes H3/H4 acetylation by Rtt109-Vps75. Asf1 equally increases the Rtt109-Vps75 specificity for both of these residues with a maximum stoichiometry of 1:1 (Asf1 to H3/H4), but does not alter the selectivity between these two residues. These data suggest that the H3/H4-Asf1 complex is a substrate for Rtt109-Vps75 without altering selectivity between residues. The deletion of either Rtt109 or Asf1 *in vivo* results in the same reduction of H3K9 acetylation, suggesting that Asf1 is required for efficient H3K9 acetylation both *in vitro* and *in vivo*. Furthermore, we found that the acetylation preference of Rtt109-Vps75 could be directed to H3K56 when those histones already possess modifications, such as those found on histones purified from chicken erythrocytes. Taken together, Vps75 and Asf1 both enhance Rtt109 acetylation for H3/H4, although via different mechanisms, but have little impact on the residue selectivity. Importantly, these results provide evidence that histone chaperones can work together via interactions with either the enzyme or the substrate to more efficiently acetylate histones.

## Introduction

Rtt109 (KAT11), a fungal-specific lysine acetyltransferase (KAT) that shares structural homology to mammalian p300/CBP [1], was first identified along with Asf1 for its critical role in the acetylation of H3K56 [2]. In addition to H3K56 acetylation, Rtt109 catalyzes the acetylation of several other residues on histone H3 and H4 [3–5]. H3K56 acetylation is unique because it occurs in the globular domain of the histone, suggesting this histone acetylation mark is also directly related to DNA accessibility and transcriptional activation [6–9]. Consistent with this, Rtt109 has been shown to be important for cell survival after exposure to a variety of genotoxic agents [10, 11]. The acetylation of H3K56 requires the histone chaperone Asf1, and the loss of Asf1 will result in a complete loss of K56 acetylation [4].

In general, histone chaperone proteins are highly acidic proteins that directly bind to histones, and guide assembly and disassembly of nucleosomes [12, 13]. Rtt109 has been reported to be activated by two different histone chaperones: Vps75 and Asf1 [14–17]. Rtt109 activation by Vps75 is via direct complexation [4, 18, 19], but it is not clear how Asf1 activates Rtt109. One possible mechanism is via the ability of Asf1 to selectively bind histone H3/H4 [20] and/or split the tetramer (H3/H4)_2_ into the dimer 2(H3/H4), thus increasing the available H3/H4 by 2-fold [21, 22].

Rtt109-chaperone complex can acetylate multiple lysines on a single histone, and it has been suggested that Asf1 can alter the selectivity of the residues acetylated [3–5]. The ability of Rtt109 to acetylate multiple residues on a single complex presents problems in understanding its specificity. To fill this gap, we utilize a novel label-free mass spectrometry-based technique that can detect individual lysine acetylation on histone H3 and H4 simultaneously [23, 24]. We have previously used this assay to investigate the differences in specificity and selectivity between CBP and p300 [24] as well as to characterize the specificity of Gcn5 [23].

Specificity refers to the ability of an enzyme to acetylate a specific residue, whereas selectivity pertains to the ability of an enzyme to acetylate one residue relative to another. It has been reported that Rtt109-Vps75 specifically acetylates H3K9 and H3K23, whereas Rtt109-Asf1 is reported to favor the acetylation of H3K56 *in vitro* [5]. Additionally, Rtt109-Asf1 suppresses acetylation at other sites on H3 and H3/H4, compared to Rtt109-Vps75, thereby altering the selectivity [5]. Understanding how these chaperones differentially regulate Rtt109 is therefore important for determining how KAT activity is controlled in the cell.

In this paper, we investigated the specificity and selectivity of acetylation by Rtt109-Vps75 and the influence of Asf1 on this specificity/selectivity. In addition, we determined how specificity changes when transitioning from a substrate of H3 to H3/H4 tetramer, in the presence or absence of Asf1. Here we demonstrated that under steady-state conditions, Rtt109-Vps75 complex primarily targets H3K9 and H3K23, both in the context of the H3/H4 tetramer and on H3 alone. Histone H3 alone was the preferred substrate of Rtt109-Vps75, and demonstrated a higher selectivity for H3K9 over H3K23. The formation of H3/H4 tetramer lowered the specificity of Rtt109-Vps75 and caused the loss of selectivity between K9 and K23. The conversion of H3/H4 tetramer to H3/H4-Asf1 substrate led to an increase in specificity [14, 25], but, interestingly, did not change the selectivity. These results provide important insight into how histone acetylation by Rtt109 is regulated, as well as the factors that influence Rtt109-Vps75 specificity/selectivity.

## Materials and Methods

### Reagents

All chemicals were purchased from Sigma-Aldrich (St. Louis, MO) or Fisher (Pittsburgh, PA) and were either the highest commercial grade or LC/MS grade. Ultrapure water was generated from a Millipore Direct-Q 5 ultrapure water system (Bedford, MA). Acetyl-CoA was obtained from Sigma-Aldrich.

### Protein preparation and purification

Recombinant Xenopus and yeast histones H3 and H4 were purified and provided by the Protein Purification Core at Colorado State University. Histone H3/H4 refolding/tetramerization was done using previously reported methods [26, 27]. Native histone H3/H4 tetramer was purified from chicken erythrocytes and provided by Dr. Studitsky’s Lab (Fox Chase Cancer Center, Philadelphia, PA). Single-site acetylated recombinant Xenopus H3/H4 tetramers (H3K9ac/H4 and H3K23ac/H4) were provided by Dr. Fierke’s Lab (Univerisity of Michigan, Ann Arbor, MI). Asf1 and Rtt109-Vps75 complex were expressed and purified following previously described procedures [3, 28, 29]. All purified protein and histone concentrations were determined by UV absorbance and calculated from the extinction coefficients [30–32]. The H3/H4-Asf1 complex was prepared by mixing H3/H4 and Asf1 to the designated molar ratios and then pre-incubating at room temperature for 30 minutes.

### KAT assays

Steady-state (E<<S) assays for recombiant Xenopus H3, H3/H4, and H3/H4-Asf1 were performed under identical buffer conditions (100 mM ammonium bicarbonate and 50 mM HEPES buffer (pH 7.8)) at 37°C. The assays contained 0.02 to 0.5 μM Rtt109-Vps75 (varied with substrate concentration) and either 0.3–40 μM H3, H3/H4, or H3/H4-Asf1 with saturating acetyl-CoA, or 0.5–500 μM acetyl-CoA with saturating histone. In addition, nonenzymatic experiments were conducted under the aforementioned buffer conditions in the absence of Rtt109-Vps75, with either 2.5 μM H3 or 2.5 μM H3/H4 and 200–500 μM acetyl-CoA. We also carried out a time course study (triplicates) to understand the site-specific details of Rtt109-Vps75 acetylation on recombiant Xenopus H3/H4. The experiemental conditions were 2 μM H3/H4, 200 μM acetyl-CoA, and 5 μM Rtt109-Vps75 at 37°C. For the acetylation assay comparing various histones (recombiant yeast and Xenopus histones and native chicken histones from erythrocytes), 15 μM H3/H4, 200 μM acetyl-CoA, and 0.43 μM Rtt109-Vps75 were added and incubated at 37°C for 1 h. These two assays were used to characterize the acetylation patterns of individual lysines under non-steady-state conditions. Sample preparation, including reaction quench, post-reaction propionylation, and tryptic digestion, were performed as in our previous published procedures [23, 33]. In order to reflect the fact that one Rtt109 molecule can only catalyze one molecular histone H3 at a time, as well as to easily compare the acetylation kinetic behavior with histone H3 alone, we use the H3/H4 dimer concentration to represent the concentrations of H3/H4, regardless of the conformation and complex.

### Stoichiometry

We determined the recombiant Xenopus H3/H4 and Asf1 stoichiometry by titrating Asf1 and maintaining saturated H3/H4 and acetyl-CoA (25 and 300 μM, respectively) under steady-state conditions (0.3 μM Rtt109-Vps75). The H3/H4 and Asf1 mixture was prepared as described above and H3/H4 concentration was at least ten-fold higher than the dissociation constant (K_d_) [15]. The apparent catalytic constant (k_cat(app)_) was plotted as a function of the molar ratio of Asf1:H3/H4. Under these conditions, the protein ratio at which the k_cat(app)_ reaches its plateau was determined to be the stoichiometry.

### Sample analysis via UPLC-MS/MS

A Waters Acquity H-class UPLC (Milford, MA) coupled with a Thermo TSQ Quantum Access (Waltham, MA) triple quadrupole mass spectrometer (MS) was used to quantify the acetylated lysines on H3 and H4 peptides. The UPLC and MS/MS settings, solvent gradient, detailed mass transitions, and peak identification/quantification were reported in our previously published work [23, 24, 33]. Retention time and specific mass transitions were both used to identify individual acetylated and propionylated peaks. The resolved peak integration was done using Xcalibur software (version 2.1, Thermo). Relative quantitative analysis was used to determine the amount of acetylation on individual lysines [23, 33–35].

### Data analysis

All the data were fit with Prism (version 5.0d). The initial rates (v) of acetylation were calculated from the linear increase in acetylation as a function of time prior to a total 10% acetylation. For individual lysines, the steady-state parameters k_cat(app)_, K_(app)_ (*i*.*e*. K_m(app)_ or K_1/2(app)_), and Hill coefficient (nH) were determined by fitting eq. 1, where [S] is the concentration of substrate (H3, H3/H4, H3/H4-Asf1, or acetyl-CoA), and [E] is the concentration of Rtt109-Vps75. In the case of multiple competitive sites with comparable acetylation rates and the same Hill coefficient, a one-site model is suitable to determine the specificity of each site, since the value of the specificity constant (k_cat(app)_/K_m(app)_ or k_cat(app)_/K_1/2(app)_^nH^) is conserved for individual sites [23]. In addition, in cases where histones are not limiting, the k_cat(app)_ is a viable measure for investigating the changes of enzyme specificity.

$$\;\frac{v}{\left[E\right]}\;=\;k_{cat(app)}\frac{{\left[S\right]}^{nH}}{\left({\left[S\right]}^{nH}+K_{\left(app\right)}^{nH}\right)}$$ eq. 1

To determine the second order rate constant (k_nE_) for nonenzymatic acetylation under the conditions of excess acetyl-CoA, nonenzymatic acetylation was described by a single exponential equation (eq. 2), where [P]_t_ is the concentration of a specific acetylated lysine at an individual sampling time point, t is the time, and k_obs_ is the observed rate.

$$\;{\left[P\right]}_{t}\;=\;{\left[P\right]}_{\infty }\left(1-e^{{-k}_{obs}t}\right)$$ eq. 2

The second order rate constant for nonenzymatic acetylation was determined by eq. 3, where k_obs_ is the pseudo-first order rate constant of acetylation from eq. 2, [acCoA] is the concentration of acetyl-CoA, k_nE_ is the second order rate constant, and b is the y-intercept.

$$k_{obs}\;=\;k_{nE}\left[acCoA\right]+b$$ eq. 3

The catalytic proficiency ((k_cat_/K_m_)/k_nE_) was used to represent the ability of an enzyme to enhance acetylation. In addition, the ΔΔG for specific lysine residue was calculated using eq. 4, where R is the gas constant, T is the absolute temperature, (k_cat_/K_m_) is the specificity constant for the residue of interest, and k_nE_ is the second order rate constant for nonenzymatic acetylation. Using ΔΔG, it is easier to recognize the enzyme preferences on different sites, because the specificity and selectivity could be quantified [23].

$$\Delta \Delta G\;=\;-RT\;ln\left(\frac{k_{cat}/K_{m}}{k_{nE}}\right)$$ eq. 4

### Yeast histone extraction

The yeast histone purification is based on established protocols with minor modifications [36]. Briefly, yeast BY4741 (wt), *asf1Δ*, and *rtt109Δ* strains were cultured in YPD (1% yeast extract, 2% peptone and 2% glucose) and collected at OD600 of ~1.0 by centrifuging. The cell pellets were washed and incubated in DTT-Tris-HCl buffer at 30°C for 15 minutes. The cells were then digested with zymolyase at the final enzyme concentration of ~2 mg/ml for 60 to 90 minutes. After centrifugation pellets were resuspended in buffer, with the addition of H_2_SO_4_ to extract histones followed by TCA precipitation. The extracted yeast histones of these three strains were analyzed by UPLC-MS/MS.

### Western blotting

Yeast cells were grown overnight in rich media, diluted and allowed to undergo 2 cell doublings. When cultures reached an OD600 of about 1.0, cells were harvested and lysed with 2 x SDS sample buffer. Proteins were denatured at 95°C for 5 minutes. Supernatants of total lysates were used for western blotting analysis. Approximately equal amount of total proteins were resolved by 15% SDS-PAGE followed by transferring of the proteins to a nitrocellulose membrane. Primary antibodies used in this study include polyclonal rabbit anti-Histone H3 (Abcam, Cambridge, MA), anti-acetylated H3K9 (Millipore), and anti-acetylated H3K23 (Millipore); each blot was also probed with polyclonal rabbit anti-TBP (as a reference). The secondary antibody was florescence labeled IR800 goat anti-rabbit (Rockland, Gilbertville, PA). The blots were visualized with an Odyssey infrared dual laser scanning unit (LI-COR Biotechnology, Lincoln, NE).

## Results

### Identification and quantification of Rtt109-Vps75-mediated acetylation on H3/H4

**Fig 1:**
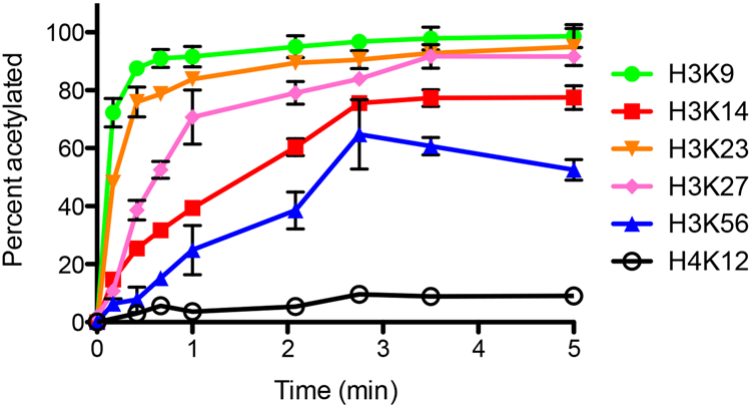
Time course study of acetylation on individual lysine residues of H3/H4 catalyzed by Rtt109-Vps75. The error bar indicates the standard error in acetylation percentage from three sets of assays. This assay indicates that H3K9 and H3K23 are the primary sites acetylated by Rtt109-Vps75, while H3K56 acetylation is modest.

We have demonstrated that this multiplex targeted MS technique can detect and quantify the extent of individual acetylated lysine residues on histone H3/H4 from one sample injection [23, 24]. Herein, we determined the extent of acetylation on individual lysine residues by Rtt109-Vps75 to understand the site preference of Rtt109-Vps75 acetylation on H3/H4 (Fig. 1). Sites where we detect less than 5% acetylation are not shown. This plot shows that Rtt109-Vps75 preferentially acetylates H3K9 and H3K23, while K14, K27, and K56 on H3 have only modest acetylation. Although Rtt109-Vps75 cannot effectively acetylate the lysines on H4 compared to H3, H4K12 is the primary acetyaltion site on H4. These acetylation patterns are in agreement with previous studies [3–5].

### Specificty of Rtt109-Vps75-mediated acetylation of H3

**Fig 2:**
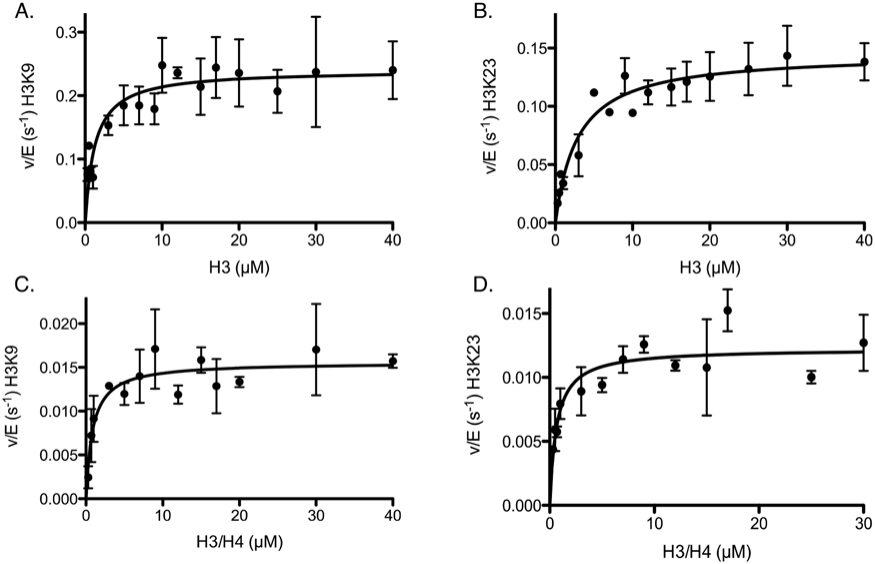
Determination of steady-state parameters for individual lysines of H3 and H3/H4 acetylation catalyzed by Rtt109-Vps75 when titrating histone in the presence of saturating acetyl-CoA. The detailed experimental conditions are described in the section “Steady-state kinetic assays for Rtt109-Vps75”. The error bar represents the standard error in v/E. Panels (A) and (B) show the Michaelis-Menten behaviors for H3K9 and H3K23, respectively, when titrating H3. Panels (C) and (D) also show the Michaelis-Menten behaviors for K9 and K23, respectively, when titrating H3/H4. H3 is the preferred substrate for Rtt109-Vps75 acetylation. The apparent kinetic parameters are summarized in Table 1.

Rtt109-Vps75 is able to acetylate multiple lysine residues on histones; however, the factors regulating the preference/order of acetylation of individual lysines remain poorly understood. While there are many factors that may influence acetylation specificity, one of these factors, different histone configurations, may play an important role in cells. Tsubota *et*. *al*. reported a k_cat_ of H3 acetylation by Rtt109-Vps75 of 0.21 sec^-1^, which is faster than that they reported for H3/H4 [18]. We hypothesized that this could be due to the fact that the structure of H3 is more dynamic, allowing greater access to multiple residues, resulting in more residues being acetylated. To address this possibility, we utilized mass spectrometry-based assays to investigate the residue specificity of H3 acetylation by Rtt109-Vps75. We identified only two residues acetylated by Rtt109-Vps75 under steady-state conditions (where the total amount of acetylation is <10%); K9 and K23 (Fig. 2A and B). The specificity (k_cat_/K_m_) of Rtt109 for residues in H3 was the highest for K9, which is ~4-fold higher than K23 (Table 1).

**Table 1:** Steady-state parameters from kinetic assays of histone titration (mean ± standard error).

Substrate Titrated	Residue Acetylated	K_(app)_(μM)	k_cat(app)_(x 10^-2^ s^-1^)	k_cat_/K_m_(x 10^-2^ μM^-1^s^-1^)
H3	H3K9	1.2 ± 0.3	24.0 ± 1.1	19.4 ± 5.2
H3	H3K23	2.9 ± 0.6	14.6 ± 0.7	5.0 ± 1.1
H3/H4	H3K9	0.9 ± 0.3	1.5 ± 0.07	1.8 ± 0.5
H3/H4	H3K23	0.6 ± 0.2	1.2 ± 0.06	1.9 ± 0.6
H3/H4-Asf1	H3K9	1.9 ± 0.5	6.2 ± 0.4	3.4 ± 1.0
H3/H4-Asf1	H3K23	2.1 ± 0.4	5.6 ± 0.3	2.6 ± 0.5

### Specificity of Rtt109-Vps75-mediated acetylation of H3/H4

Next we wanted to determine how the formation of tetramer would alter the specificity of Rtt109-Vps75 under saturating acetyl-CoA conditions. In these studies, we utilized H3 and H4 that was refolded into tetramer. However, all concentrations are listed as dimer concentrations instead of tetramer to make comparisons with data from H3 alone easier. With tetramer, we again only observed H3K9 and H3K23 acetylation under steady-state conditions. Under these conditions, the specificity for K9 was decreased by ~10-fold, and specificity for K23 was decreased by 2.5-fold when compared to H3 alone (Fig. 2C and D, Table 1). By comparing the specificity of Rtt109-Vps75 for one site to another, we obtained the selectivity of Rtt109-Vps75. We found that with the tetramer, there was no difference in selectivity between K9 and K23, which is not the case for H3 alone. Taken together, these data suggest that the order of specificity constants is H3K9 > H3K23 > H3K9/H4 ≅ H3K23/H4 (Fig. 2A-D, Table 1).

### Determining the stoichiometry of Asf1 to H3/H4 in Rtt109-Vps75 acetylation

Rtt109 is a unique KAT in that its activity is often linked to the presence of Vps75 and/or Asf1. However, the role of Asf1 in the Rtt109-mediated acetylation of H3 is not entirely clear. Several groups have suggested that Asf1 acts to alter the selectivity of Rtt109, resulting in the acetylation of H3K56 over other lysines [3–5, 14, 18]. It has be reported that Asf1 can stimulate acetylation for H3/H4 but not for H3 and that Asf1 will only form a complex with Rtt109 in the presence of H3/H4 [18]. While little work has been done utilizing both Vps75 and Asf1 together, the work that has been done suggests that they have an additive effect [14, 18]. While Vps75 interacts with the (H3/H4)_2_ tetramer, Asf1 has been shown to split the (H3/H4)_2_ tetramer into 2(H3/H4) molecules, and has a tight affinity (of ~10 nM) [18, 21, 22, 37]. Given this available data, we hypothesize that H3/H4-Asf1 could be an ideal substrate for Rtt109-Vps75.

**Fig 3:**
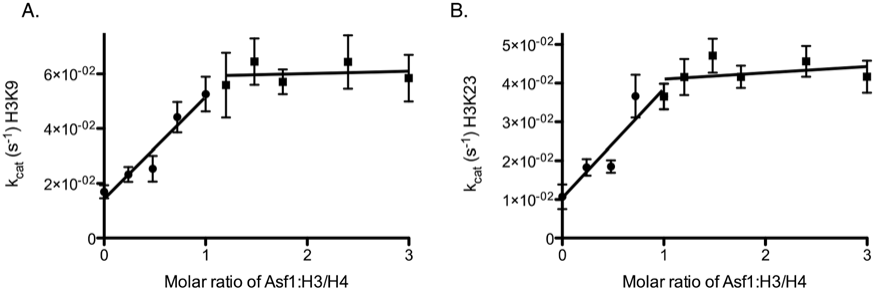
Stoichiometry of H3/H4-Asf1 for K9 and K23 in the presence of fixed concentrations of saturating acetyl-CoA and H3/H4. The detailed experimental conditions are described in the section “Stoichiometry”. The error bar represents the standard error in k_cat(app)_. Panels (A) and (B) show that the acetylation rates of H3K9 and H3K23, respectively, increase as a function of molar ratio of Asf1 to H3/H4, before all H3/H4 binds with Asf1. The inflection points of H3K9 and H3K23 are 1.21 and 1.09, respectively, indicating that the binding stoichiometry of Asf1 and H3/H4 dimer is approximately 1:1.

This hypothesis predicts that the optimal ratio of Asf1 to H3/H4 (dimer) would be 1:1 (or 1:0.5 for tetramer). To test this, we titrated Asf1 under conditions of saturating H3/H4 and acetyl-CoA (25 and 300 μM, respectively) and measured the changes of k_cat(app)_ of Rtt109-Vps75. The concentration of H3/H4 was greater than the binding affinity of H3/H4 to Asf1 (13). Under these conditions, if the complex of Asf1 with H3/H4 (H3/H4-Asf1) acts as a substrate that facilitates Rtt109-Vps75 acetylation, we should see an approximately linear increase in acetylation rate as Asf1 is increased, reaching a plateau at a ratio of 1:1. However, if Asf1 is only acting to split (H3/H4)_2_ to 2(H3/H4) without attaching to histones, we would observe maximal activation of Rtt109 acetylation at a concentration less than that of H3/H4, as Asf1 would not have to remain bound to H3/H4 after splitting the tetramer. What we in fact observed was an increase in the rate of acetylation up to a ratio of 1 Asf1 to 1 H3/H4 (K9 = 1.21 and K23 = 1.09) at which point the k_cat(app)_ starts to level off (Fig. 3A and B). These data suggest that H3/H4-Asf1 acts as a substrate for Rtt109-Vps75.

### H3/H4-Asf1 complex increases specificity but does not alter selectivity of Rtt109-Vps75

**Fig 4:**
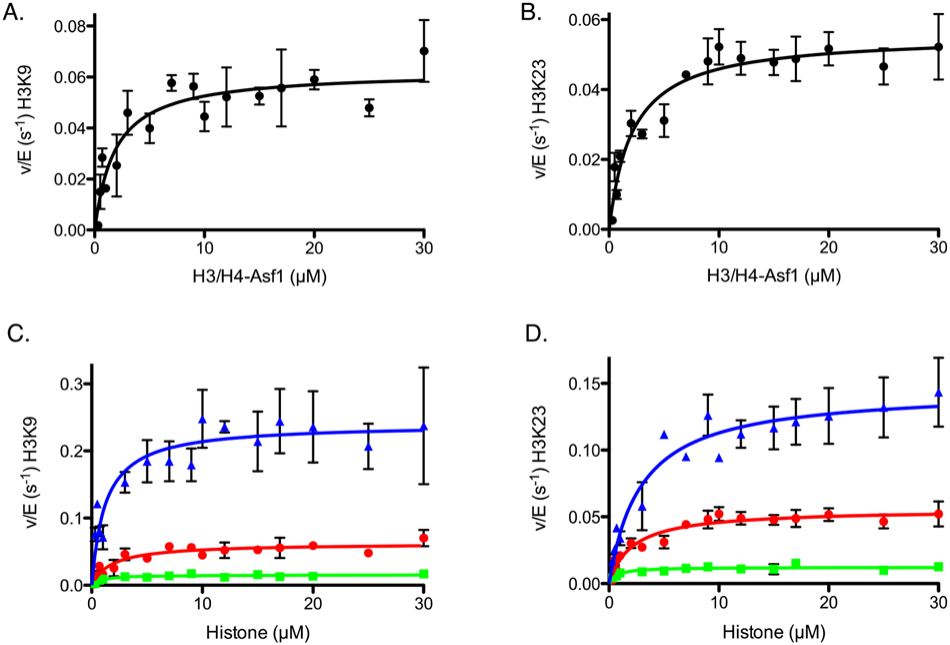
Determination of steady-state parameters of H3/H4-Asf1 acetylation catalyzed by Rtt109-Vps75 for individual lysines. The detailed experimental conditions are described in the section “Steady-state kinetic assays for Rtt109-Vps75”. The error bar represents the standard error in v/E. Panels (A) and (B) show the Michaelis-Menten behaviors for H3K9 and H3K23, respectively, when titrating H3/H4-Asf1 in the presence of saturating acetyl-CoA. Panels (C) and (D) show the superposition of steady-state kinetics for H3K9 and H3K23, respectively, on different histone conformations (H3: blue triangle, H3/H4-Asf1: red circle, and H3/H4: green square). The apparent kinetic parameters are summarized in Table 1. When H3/H4 is complexed with Asf1, no cooperativity is detected, and the specificity and selectivity of K9 and K23 acetylation increases.

Once we determined that 1:1 Asf1 to H3/H4 dimer was the optimal ratio, we wanted to determine if Asf1 had any impact on the specificity/selectivity between residues. To our surprise, we observed no additional acetylated sites other than H3K9 and H3K23 in the presence of Asf1. However, we were still interested in determining the effects of Asf1 on the specificity of Rtt109-Vps75, so steady-state assays were conducted utilizing H3/H4-Asf1 as a substrate. Assays titrating H3/H4-Asf1 were performed in the presence of saturating acetyl-CoA. The H3/H4 concentrations were maintained at a level higher than the K_d_ of H3/H4 to Asf1 and a molar ratio of 1:1.2 (H3/H4:Asf1) to ensure that all H3/H4 is bound to Asf1. We observed negligible changes in selectivity (or preference) between K9 and K23 as compared to H3/H4 alone, but, based on changes in the k_cat(app)_, we did see at least a 4-fold increase in specificity for both residues after the addition of Asf1 (Fig. 4A-D, Table 1). However, both the specificity constants and k_cat(app)_ for H3/H4-Asf1 are still less than those for H3 alone (Table 1).

### Acetyl-CoA has no impact on residue selectivity between H3K9 and H3K23 acetylation

It has been shown that acetyl-CoA concentration *in vivo* varies with different nutrient conditions, and that different acetyl-CoA concentrations could regulate histone acetylation [38–40]. Thus, after determining the different specificity on different histone substrates, we wanted to investigate if histone binding could have any impact on acetyl-CoA turnover, and whether the concentration of acetyl-CoA alters the selectivity between sites of acetylation, as it can with p300 and/or CBP [24].

**Fig 5:**
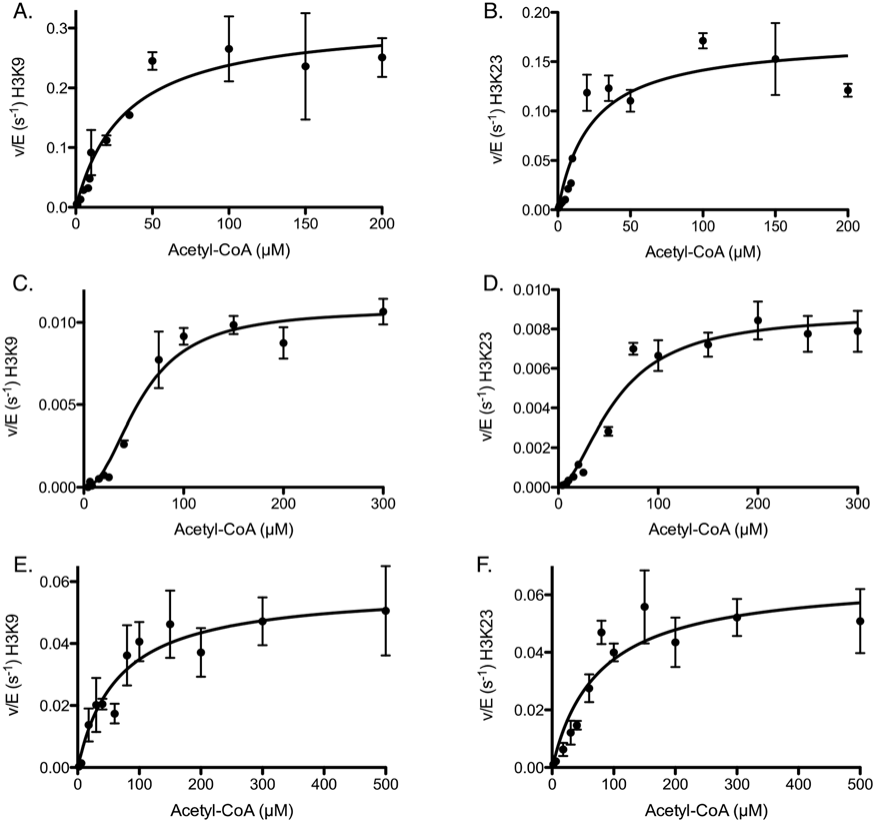
Determination of steady-state parameters for individual lysines of H3 and H3/H4 acetylation catalyzed by Rtt109-Vps75 when titrating acetyl-CoA in the presence of saturating histone. The detailed experimental conditions of the acetyl-CoA titration are described in the section “Steady-state kinetic assays for Rtt109-Vps75”. The error bar represents the standard error in v/E. Panels (A) and (B) show the Michaelis-Menten behaviors for H3K9 and H3K23, respectively. Panels (C) and (D) show the cooperativity for K9 and K23, respectively, for H3/H4. The Hill coefficient is approximately equal to 2, suggesting acetyl-CoA may have cooperative effects on the Rtt109-Vps75 (2:2) complex. Panels (E) and (F) show the Michaelis-Menten behaviors (no cooperativity) for K9 and K23, respectively, when titrating acetyl-CoA in the presence of saturating H3/H4-Asf1. The apparent kinetic parameters are summarized in Table 2.

To test this possibility, we performed a titration of acetyl-CoA with either saturating H3 or H3/H4, with the titrated concentrations of acetyl-CoA falling well within cellular acetyl-CoA concentrations [39]. When acetyl-CoA was titrated in the presence of H3, we no longer detected as large a difference in the specificity constants between K9 and K23 (Fig. 5A and B, Table 2), which is mainly due to a similar K_m_ for both K9 and K23 when acetyl-CoA is limiting. For H3/H4, we did observe a sigmoidal or cooperative (nH = 2) dependence on acetyl-CoA for both K9 and K23 (Fig. 5C and D, Table 2). While the specificity of acetylation for H3/H4 decreased ~5-fold, the selectivity remained the same between the residues.

**Table 2:** Steady-state parameters from kinetic assays of acetyl-CoA titration (mean ± standard error).

Saturating Histone	Residue Acetylated	K_(app)_(μM)	nH	k_cat(app)_(x 10^-2^ s^-1^)	k_cat_/K_m_(x 10^-2^ μM^-1^s^-1^)	k_cat_/K_1/2_^nH^(x 10^-6^ μM^-nH^s^-1^)
H3	H3K9	32.4 ± 7.7	1	31.5 ± 2.6	0.9 ± 0.2	n.a.
H3	H3K23	23.0 ± 7.3	1	17.4 ± 1.8	0.7 ± 0.3	n.a.
H3/H4	H3K9	57.6 ± 5.9	2.1 ± 0.2	1.1 ± 0.05	0.018 ± 0.002	2.2 ± 0.1
H3/H4	H3K23	57.3 ± 7.2	1.8 ± 0.1	0.9 ± 0.04	0.015 ± 0.002	6.0 ± 0.3
H3/H4-Asf1	H3K9	65.7 ± 18.0	1	5.8 ± 0.5	0.09 ± 0.03	n.a.
H3/H4-Asf1	H3K23	75.2 ± 27.0	1	6.6 ± 0.8	0.09 ± 0.03	n.a.

Finally, we sought to characterize whether the effect of acetyl-CoA on specificity is different in the presence on Asf1. As with the previous set of H3 and H3/H4 steady-state assays, we also performed acetyl-CoA titrations in the presence of saturating H3/H4-Asf1. Under saturating H3/H4-Asf1 conditions, we note that all of the titration plots from the steady-state assays of the H3/H4-Asf1 follow Michaelis-Menten kinetics (Fig. 5E and F). That indicates that there is no cooperativity when H3/H4-Asf1 is the histone substrate. Additionally, the specificity constants from the acetyl-CoA titrations were larger than those from the saturating H3/H4 condition but still smaller than those from saturating H3 alone (Table 2).

### Nonenzymatic acetylation and catalytic proficiency of Rtt109-Vps75

We have previously characterized the nonenzymatic rate of acetylation for H3 [23]. The nonenzymatic rate of acetylation can be used to calculate the catalytic proficiency of each residue [23], and the difference between catalytic proficiency for different residues is a measurement of selectivity. From the results of nonenzymatic acetylation assays, there was no significant amount of acetylation detected on any of the lysines at the longest time point for steady-state assays (0.5 h). However, at longer incubation periods, we observed multiple acetylated lysines, including H3K9, H3K14, H3K18, H3K23, and H3K64. These data are consistent with previously reported data from our lab on residue selectivity of nonenzymatic acetylation, where nonenzymatic acetylation is not random but prefers H3K36 for H3 alone [23]. Under different buffer conditions (higher pH; see Materials and Methods), we found that H3K23 was the preferred site of acetylation for both H3 and H3/H4 for nonenzymatic acetylation. Although four other sites (H3K9, H3K14, H3K18, and H3K64) were also acetylated, due to the significantly slower rates we could not obtain the second order rate constants for these other sites.

Because H3K23 was the fastest nonenzymatic acetylation site, we defined the acetylation rate of H3K23 as the upper limit of nonenzymatic acetylation, and the k_nE_ were determined from H3K23 by eq. 2 and 3 (6.9 ± 3.1 x 10^-8^ μM^-1^s^-1^ and 9.1 ± 1.4 x 10^-8^ μM^-1^s^-1^ for H3 alone and H3/H4, respectively). These data suggest that buffer conditions (ionic strength and pH) have the largest effect on the selectivity of nonenzymatic acetylation relative to histone conformation.

We determined the selectivity between K9 and K23 by calculating the catalytic proficiency of the enzyme, (k_cat_/K_m_)/k_nE_, and ΔΔG for residues K9 and K23 based on eq. 4 (Tables 3 and 4). The calculation of (k_cat_/K_m_)/k_nE_ for H3/H4-Asf1 is based on the k_nE_ from H3/H4. For titrations of the histone, there was a 2.0 x 10^5–^2.8 x 10^6^ fold enhancement with Rtt109-Vps75 catalysis (Table 3). This range accounts for the fact that different histone states or complexes resulted in different levels of catalytic enhancement. H3 demonstrates the highest catalytic enhancement of acetylation, H3/H4 the least and H3/H4-Asf1 is only slightly higher than H3/H4. Of the sites in H3, K9 was the preferred site catalyzed by Rtt109-Vps75, and there was no residue differences betweeen K9 and K23 in either H3/H4 configuration.

**Table 3:** Catalytic proficiency of Rtt109-Vps75-mediated acetylation from histone titration (mean ± standard error).

Histone Titrated	Residue Acetylated	(k_cat_/K_m_)/k_nE_(No unit)	ΔΔG (kcal/mol)
H3	H3K9	(2.8 ± 1.5) x10^6^	-9.1 ± 0.3
H3	H3K23	(7.3 ± 3.7) x10^5^	-8.3 ± 0.3
H3/H4	H3K9	(2.0 ± 0.7) x10^5^	-7.5 ± 0.2
H3/H4	H3K23	(2.1 ± 0.7) x10^5^	-7.5 ± 0.2
H3/H4-Asf1	H3K9	(3.7 ± 1.2) x10^5^	-7.9 ± 0.2
H3/H4-Asf1	H3K23	(2.9 ± 0.8) x10^5^	-7.7 ± 0.2

**Table 4:** Catalytic proficiency of Rtt109-Vps75-mediated acetylation from acetyl-CoA titration (mean ± standard error).

Saturating Histone	Residue Acetylated	(k_cat_/K_m_)/k_nE_(No unit)	(k_cat_/K_1/2_^nH^)/k_nE_ (μM^-nH+1^)	ΔΔG (kcal/mol)
H3	H3K9	(1.4 ± 0.7) x10^5^	n.a.	-7.3 ± 0.3
H3	H3K23	(1.1 ± 0.6) x10^5^	n.a.	-7.1 ± 0.4
H3/H4	H3K9	(2.1 ± 0.4) x10^3^	(2.4 ± 0.4) x10^1^	n.a.
H3/H4	H3K23	(1.7 ± 0.3) x10^3^	(6.6 ± 1.0) x10^1^	n.a.
H3/H4-Asf1	H3K9	(9.7 ± 3.2) x10^3^	n.a.	-5.6 ± 0.2
H3/H4-Asf1	H3K23	(9.6 ± 4.0) x10^3^	n.a.	-5.6 ± 0.3

For the acetyl-CoA titration, we observed a 1.7 x 10^3–^2.4 x 10^5^ fold acetylation enhancement by Rtt109-Vps75 (Table 4). However, if the Hill coefficient is taken into account, the value of (k_cat_/K_m_) should be replaced by (k_cat_/K_1/2_^nH^), and ΔΔG cannot be obtained from eq. 4 (for details see [23]). A Hill coefficient of ~2 results in about a two-order of magnitude decrease in catalytic proficiency for the H3/H4 data but does not change the order of selectivity between the residues or affect which histone state is preferred as a substrate. In all conditions, H3 is the most catalytically proficient, and acetyl-CoA had no impact on the selectivity between H3K9 and H3K23.

### *In vivo* loss of acetylation due to the loss of either Rtt109 or Asf1

**Fig 6:**
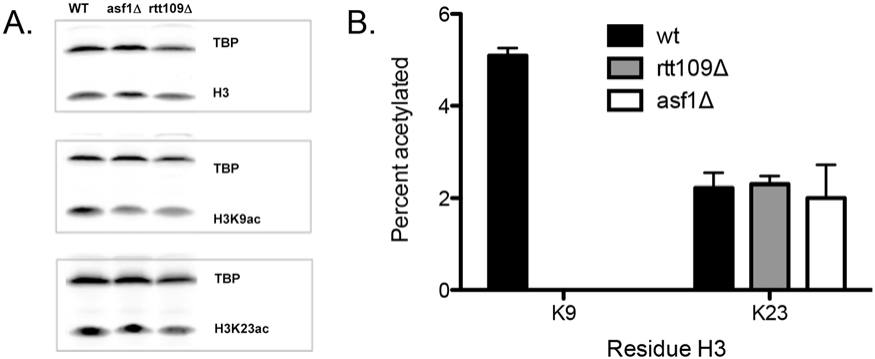
Changes *in vivo* due to the loss of either Rtt109 or Asf1. (A) Western blots using antibodies for H3, H3K9 or H3K23, using whole cell extracts from wild type (wt), *rtt109Δ*, and *asf1Δ* strains. (B) Determination of the percentage of acetylation at position H3K9 or H3K23 in wt, *rtt109Δ*, and *asf1Δ* strains using the targeted MS-based method. The error bar represents the standard error in acetylation percentage.

Several labs have examined the loss of acetylation due to the loss of either Rtt109 or Asf1 [3, 4, 16, 25, 41], but the focus of these reports has largely been on the acetylation of H3K56. Western blots demonstrated a loss of H3K9 that is consistent with results from Berndsen *et*. *al*. [3], but we have not detected a significant decrease in H3K23 acetylation (Fig. 6A). As it is difficult to rule out issues related to using antibodies (e.g. cross reactivity and epitope occlusion), we chose to use our targeted MS approach to monitor histone acetylation in these mutations. In the present study, we grew each strain in duplicate and preformed three individual sample injections from each sample. From our MS analysis, we observed at least a 5% decrease in acetylation of H3K9 with the loss of either Rtt109 or Asf1, while there are no significant changes of acetylation detected on H3K23 from these two mutants (Fig. 6B), which is consistent with our observations from western blot analysis. If we assume that in the cell H3 mainly exists in complex with H4, then these data suggest that Asf1 is needed for optimal H3K9 acetylation by Rtt109, both *in vitro* and *in vivo*.

The results of our analyses indicate that Rtt109 is one of the essential KATs for H3K9 acetylation but not for H3K23 acetylation *in vivo*. This finding could be due to the fact that H3K9 has been proposed to be acetylated by only Rtt109 and Gcn5 in yeast [4], while H3K23 is likely to be acetylated by multiple KATs. In addition, the deletion of Asf1 results in the same amount of K9 acetylation loss as the deletion of Rtt109, suggesting Asf1 is necessary for Rtt109 acetylation *in vivo*.

### Factors influencing H3K56 acetylation by Rtt109-Vps75

**Fig 7:**
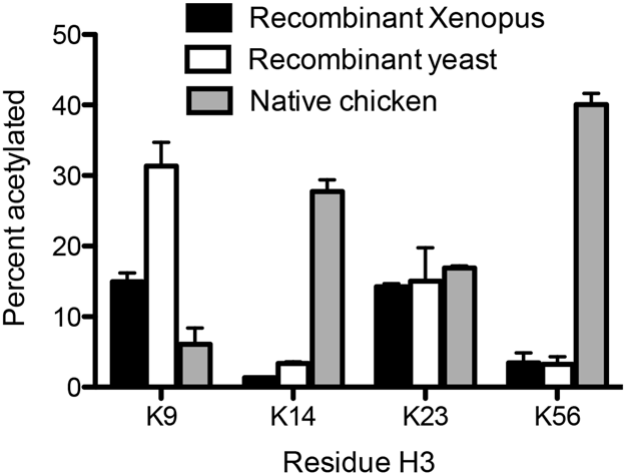
Comparison of Rtt109-Vps75 mediated acetylation (1-h incubation) on individual lysines on H3 from chicken erythrocytes, recombinant Xenopus histones, or recombinant yeast histones. Acetylation percentage of each site was determined by targeted MS-based method. The black and white bars depict the acetylation levels on recombinant Xenopus and yeast histones, respectively. The grey bar represents the acetylation levels on native chicken H3/H4 after 1 hour incubation with Rtt109-Vps75. The error bar represents the standard deviation in acetylation percentage from triplicate measurements. Note that the acetylation amount on both recombinant histones is initially negligible; however, there is ~25% H3K14ac and ~12% H3K23ac preexisting acetylation on chicken histones.

Rtt109 was originally identified for its ability to support the acetylation of H3K56 [2, 42, 43], but our data indicate that this is not its primary or initial site of acetylation under our experimental conditions. We identified one possible difference: the use of histones purified from chicken erythrocytes [44]. We therefore wanted to determine if these different histone substrates combined with longer incubation time are key to facilitating H3K56 acetylation. Before utilizing histones from chicken erythrocytes, we used our label-free MS-based method to identify whether any residues were already acetylated, prior to acetylation by Rtt109-Vps75. We detected preexisting acetylation on histones from chicken erythrocytes of ~25% H3K14ac and ~12% H3K23ac, while there is no significant acetylation found on any other lysine residues of chicken erythrocyte histones. We also determined that no lysines from recombinant yeast nor Xenopus histones were acetylated prior to our experiments. Additionally, it is likely that other modifications exist on the chicken erythrocyte histones (such as methylation or phosphorylation) that we did not detect in this assay. Therefore, chicken histones present a complex background that is not suitable for kinetic studies. However, utilizing the chicken histones allows us to estimate the impact of pre-modified histones. Thus, we incubated 15 μM H3/H4 (recombinant yeast, Xenopus histones or histones purified from chicken erythrocytes) and 0.43 μM Rtt109-Vps75 (about the same level used for steady-state assays) for a longer time (1h). Under these conditions, we detected modest acetylation of K56, on both recombinant histones. However, the major site of acetylation on native chicken histone was H3K56 (Fig. 7). Although there were slight differences in the extent of acetylation between recombinant Xenopus and yeast histones, the preferences of Rtt109-Vps75 acetylation remained the same. The requirement for additional post-translational modification(s) presents a potential cause for this alteration in the specificity/selectivity of Rtt109-Vps75 towards K56 acetylation.

## Discussion

Rtt109-Vps75 targets lysines on histones that are involved in genome stability and nucleosome assembly. In order to understand the extent to which Rtt109-Vps75 targets specific lysines in histone, we used a label-free quantitative MS-based assay to determine the specificity and selectivity of Rtt109-Vps75 on individual lysines of different histone substrates. In doing so, we have expanded our understanding of how chaperones help to facilitate Rtt109 dependent acetylation.

Much of the previously published work on Rtt109 has been done using classical techniques that either quantitate the total amount of acetylation or identify the locations of acetylation, but not both. Because of this limitation, the investigation of the specificity/selectivity of this enzyme is incomplete. Targeted MS can overcome this problem, allowing us to accurately measure the increases in catalytical activity and/or the changes in selectivity for individual sites.

We have previously shown with p300 that the histone configuration (H3 or H3/H4) can affect the specificity of a KAT. Here we observed that H3 was a much better substrate for Rtt109-Vps75 than H3/H4. We also observed a higher specificity for K9 and K23 on histone H3 alone, compared to H3/H4. Rtt109-Vps75 prefers K9 and K23 to other reisidues in all cases, with H3 being the preferred substrate *in vitro*. While *in vivo* a majority of H3 is bound to H4 to form either a histone-histone chaperone complex or a nucleosome, it cannot be ruled out that H3 is a possible substrate *in vivo*. Furthermore, the role of H3K9 acetylation in newly produced histones and their deposition in yeast [45], could suggest that Rtt109-Vps75 plays an important role in histone deposition of newly synthesized histone H3.

We observed no significant difference in selectivity between K9 and K23 on different histone configurations with decreasing acetyl-CoA, which suggests that reduced levels of acetyl-CoA in the cell will not affect the selectivity between these two primary sites acetylated by Rtt109-Vps75. In addition, when titrating acetyl-CoA, the cooperativity (nH = 2) was only observed in the presence of saturating H3/H4, but not when titrating H3/H4 in saturating acetyl-CoA. One possible explanation for this could stem from the fact that the Rtt109-Vps75 complex is reported to be a 2:2 complex [15, 17]. It is also known that one acetyl-CoA binds to one Rtt109, which then allows Rtt109 to acetylate histones. At low levels of acetyl-CoA, it is more likely that only one of the two Rtt109 molecules in a complex with Vps75 dimer may have an acetyl-CoA molecule bound, and Vps75 dimer will bind one H3/H4 tetramer [15]. This will result in only one of the two H3 molecules being acetylated and thus the reduced acetylation efficiency. As the acetyl-CoA concentration increases, the probability of both Rtt109 molecules in complex having an acetyl-CoA molecule bound increases, and thus there is a non-hyperbolic increase in v/E as a function of acetyl-CoA. This model also explains why cooperativity is not observed in H3 monomer titrations.

In addition to Vps75, Asf1 has been suggested to alter the residues selectivity of Rtt109 [4, 5, 14, 18]. Asf1 interacts with H3/H4 dimer instead of (H3/H4)_2_ tetramer [21, 22, 37]. In the present study, we followed up on the possibility that Asf1 binds H3/H4 dimer and functions as a substrate for Rtt109-Vps75. Tsubota *et*. *al*. reported that H3/H4 is required for interactions with Asf1 and increases the apparent acetylation rate of Rtt109-Vps75 [18]. Consistent with this finding, we observed a 1:1 stoichiometry of Asf1 to H3/H4 dimer and 4~5-fold increases in the k_cat(app)_. Further supporting a model where H3/H4-Asf1 is acetylated by Rtt109-Vps75 is the fact that when we titrated acetyl-CoA under saturating H3/H4-Asf1, we lost the cooperativity observed in the H3/H4 alone reactions. Given the current structural model of 2:2 Rtt109:Vps75 and this loss of cooperativity, it suggests that H3/H4-Asf1 interaction with Rtt109-Vps75 increases the rate of acetylation. Therefore, Asf1 may enhance Rtt109-Vps75 activity by correctly positioning the histone substrate in the primed Rtt109 active site with acetyl-CoA.

While it has previously been demonstrated that the total acetylation of H3/H4 is enhanced by the introduction of Asf1 to Rtt109-Vps75 *in vitro* [14, 25], the analyses of each of the individual lysines have not been reported due to the aforementioned limitations of detection. Han and coworkers reported that H3K56 acetylation is enhanced by adding Asf1 into Rtt109-Vps75 and H3/H4 [14], and our data demonstrate that Asf1 enhances Rtt109-Vps75 acetylation on H3K9 and HK23 without altering the selectivity or preference between possible sites. This would mean that even if K56 acetylation has been increased, but is still a secondary site, we would not detect significant acetylation on K56 prior to 10% acetylation of the primary sites. We also conclude that an increase in specificity due to additional Asf1 is from a significant increase in k_cat(app)_ and to a lesser degree K_(app)_ (Tables 1 and 2). This finding is consistent with the study of Rtt109-Vps75 and H3/H4-Asf1 in which no significant enhancement in binding is observed [25], although it is unclear how the spatial arrangement of this H3/H4-Asf1 complex could influence the reaction rate. Thus, we suggest that Vps75 and Asf1 work together to promote histone acetylation in the cell. Consistent with this result, we observed the same amount of decrease in K9 acetylation *in vivo* with the loss of either Asf1 or Rtt109, suggesting that both are needed for proper acetylation.

Preexisting modification patterns are capable of altering the acetylation pattern of Rtt109-Vps75, as seen using histones extracted from chicken erythrocytes. Chicken erythrocyte histones were the only factor capable of altering the selectivity such that Rtt109-Vps75 preferentially acetylated H3K56. However, preexisting histone modifications unfortunately confound the results, because there is no one modification to which the altered selectivity can be attributed. Further evidence that preexisting modifications can alter selectivity is the fact that we observed suppression of K9 and K23 acetylation when utilizing chicken erythrocyte histones. This suggests that preexisting modifications on the histones have a bigger influence on residue selectivity than histone chaperones. While K56 acetylation is likely to occur secondarily to K9 and K23 acetylation no matter what the concentrations of Rtt109-Vps75 are, K56 acetylation may not be dependent on K9 and K23 acetylation, but instead is simply less favorable. This idea is supported by the fact that native chicken histones have modest H3K9 and H3K23 acetylation by Rtt109-Vps75 but significant enhancement of K56 acetylation (Fig. 7). However, under steady-state conditions using histones with no preexisting marks, Rtt109-Vps75 is not capable of acetylating K56 efficiently. In addition, we utilized either H3K9ac/H4 or H3K23ac/H4 tetramer as a starting substrate for 1-h incubation with Rtt109-Vps75 (same experimental condition of the assay for Fig. 7). After 1 hour, K56 of H3K9ac/H4 or H3K23ac/H4 tetramer were both ~3.9% acetylated, which is similar to the moderate acetylation of K56 detected from unmodified recombinant Xenopus and yeast histone tetramer (3.4 ± 1.4% and 3.2 ± 1.1%, respectively). Because of the high level of K56 acetylation detected when utilizing native chicken histones, these data suggest that some initial post-translational modification(s), but not K9 and K23 acetylation, alters the specificity and selectivity of Rtt109-Vps75 to favor K56 acetylation.

These data highlight that histone conformations (monomer, tetramer, and histone-chaperone complex) have significant effects on residue specificity for enzymatic acetylation. This suggests that chromatin dynamics play an important role in the specificity of KATs, linking both aspects of histone chaperones, which can both alter chromatin dynamics and histone conformation, which in turn influences acetylation. In this paper, we present evidence of increased specificity by two different chaperones interacting with different proteins: Rtt109-Vps75 and H3/H4-Asf1 working together for optimal acetylation. Finally, together these data demonstrate that a label-free quantitative mass spectrometry-based assay is a useful tool to facilitate the understanding of enzyme kinetics, and provide a better understanding of protein-chaperone interactions and their role in histone acetylation.
